# Mass spectrometry-based metabolomics for discovering active ingredients and exploring action mechanism of herbal medicine

**DOI:** 10.3389/fchem.2023.1142287

**Published:** 2023-03-31

**Authors:** Sifan Guo, Shi Qiu, Ying Cai, Zhibo Wang, Qiang Yang, Songqi Tang, Yiqiang Xie, Aihua Zhang

**Affiliations:** ^1^ International Advanced Functional Omics Platform, Scientific Experiment Center and Hainan General Hospital, College of Chinese Medicine, Hainan Medical University, Haikou, China; ^2^ Graduate School, Heilongjiang University of Chinese Medicine, Harbin, China

**Keywords:** Metabolomic, mass spectrometry, herbal medicine, target, biomarker, mechanism

## Abstract

Natural products derived from herbal medicine are a fruitful source of lead compounds because of their structural diversity and potent bioactivities. However, despite the success of active compounds derived from herbal medicine in drug discovery, some approaches cannot effectively elucidate the overall effect and action mechanism due to their multi-component complexity. Fortunately, mass spectrometry-based metabolomics has been recognized as an effective strategy for revealing the effect and discovering active components, detailed molecular mechanisms, and multiple targets of natural products. Rapid identification of lead compounds and isolation of active components from natural products would facilitate new drug development. In this context, mass spectrometry-based metabolomics has established an integrated pharmacology framework for the discovery of bioactivity-correlated constituents, target identification, and the action mechanism of herbal medicine and natural products. High-throughput functional metabolomics techniques could be used to identify natural product structure, biological activity, efficacy mechanisms, and their mode of action on biological processes, assisting bioactive lead discovery, quality control, and accelerating discovery of novel drugs. These techniques are increasingly being developed in the era of big data and use scientific language to clarify the detailed action mechanism of herbal medicine. In this paper, the analytical characteristics and application fields of several commonly used mass spectrometers are introduced, and the application of mass spectrometry in the metabolomics of traditional Chinese medicines in recent years and its active components as well as mechanism of action are also discussed.

## 1 Introduction

Exploring the safety and effectiveness of herbal medicine, such as traditional Chinese medicine (TCM), is the key to its modernization and internationalization (Zhang et al., 2011; Ren et al., 2023). It is of great practical and long-term significance to excavate and utilize TCM resources for the development of China’s pharmaceutical industry (Wang X. et al., 2012; Zhang et al., 2012a). Especially in the large epidemic situation of novel coronavirus infection in the past 2 years, the advantages and characteristics of TCM are increasingly emerging. However, like almost all traditional medicine, TCM also faces severe challenges (Zhang et al., 2018a). The lack of scientific and technological means and the lack of modern research restrict the development of TCM worldwide (Zhang et al., 2012b). Therefore, building a communication bridge between TCM and modern medicine has become an important issue in the field of modern biological sciences (Zhang et al., 2010). The advent of metabolomics (Johnson et al., 2016) has gradually made the clear link between TCM and modern medicine. Metabolomics has become more prevalent in TCM research during the past few years, such as in cancer (Lv et al., 2019; Chen et al., 2020; Li et al., 2020), cardiovascular disease (Mcgarrah et al., 2018; Li et al., 2019; Wang X. et al., 2020), senile dementia (Zhang et al., 2018b; Wei et al., 2019; Yi et al., 2020), liver injury (Zhang et al., 2013a; Li et al., 2017; Wei et al., 2020), etc. The application of metabolomics to understand the process of action of TCM is consistent with the overall syndrome differentiation observation of TCM (Wang et al., 2017).

**FIGURE 1:**
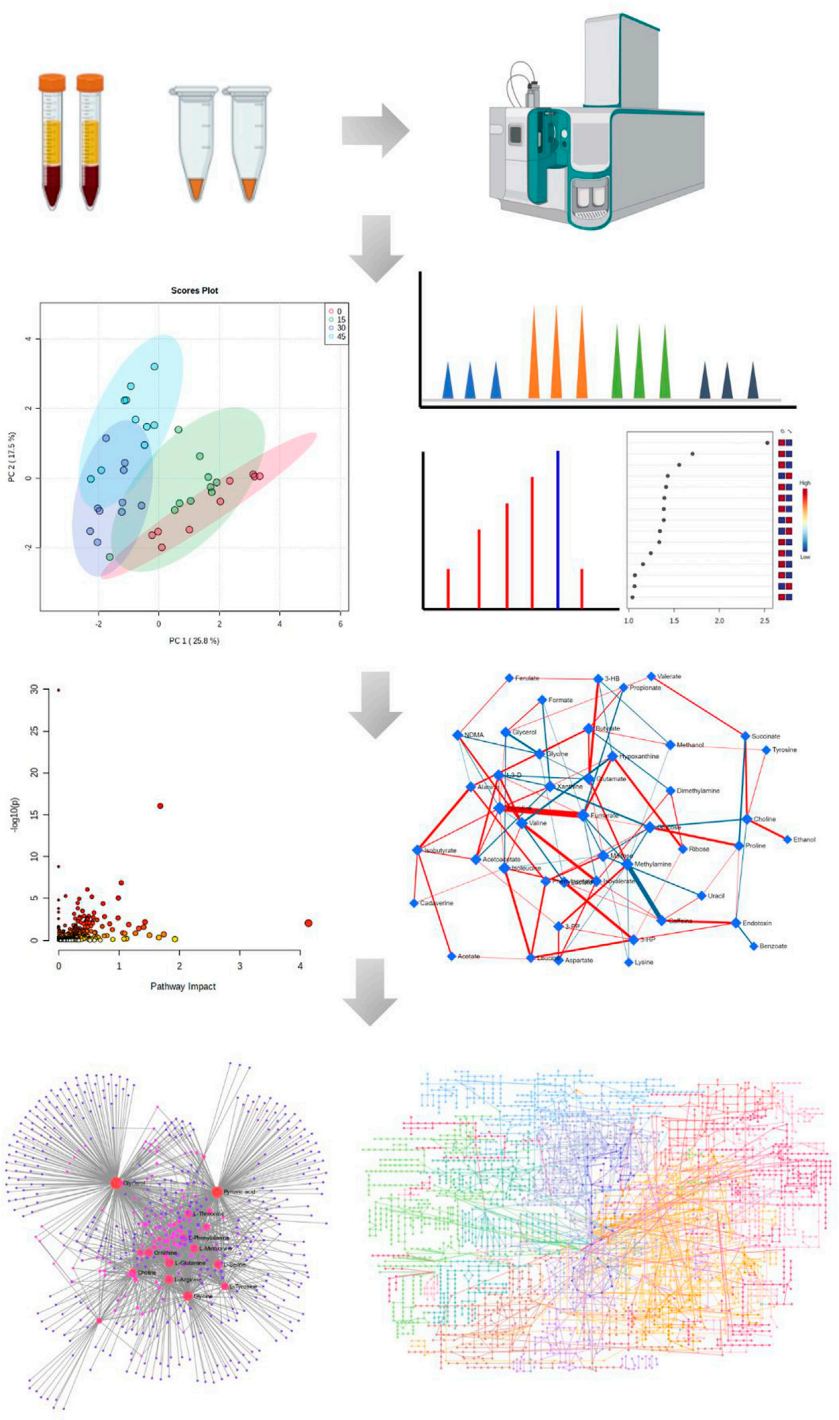
The experiment workflow of metabolomics. All images were obtained using the example data provided by the MetaboAnalyst 5.0 and figures created by BioRender.

Terminal downstream products of the genome are known as the metabolome (Zamboni et al., 2015), which is composed of metabolites of all low-molecular-weight molecules in cells, tissues, or organisms, and the metabolome possesses a lot of information that is thought to best predict the phenotype. Metabolomics uses modern instrumental analysis methods, combined with pattern recognition, to analyze metabolite changes over time after a biological system is stimulated or disturbed (Schrimpe-Rutledge et al., 2016). In biochemistry, metabolomics is the ultimate end-point measure of biological events that link genotype to phenotype but also captures the effects of nutrition, environmental influences, drug response, etc. The importance of metabolomics in assessing health and treatment impact is also manifested in biological timing and biological probability: Genetics and genomics capture possible events. Proteomics captures ongoing events, and metabolomics captures events that have occurred (Amberg et al., 2017). In addition, due to the intrinsic sensitivity of metabolomics, small modifications in metabolic mechanisms and internal environments of organisms can be discovered to shed light on the mechanisms underlying a variety of physiological situations and abnormal phenomena (Johnson et al., 2016). Metabolomics was originally proposed as an approach to functional genomics (Przybyla and Gilbert, 2022), but its use is far more than that. It contributes greatly when changes in metabolite levels need to be assessed. The main advantage is that metabolomics methods can specifically compare the changes in the metabolic profile of the human body, elucidate the mechanism of action and action changes of TCM, make up for the lack of single components and single targets of TCM research methods, and provide a more scientific and reasonable comprehensive explanation for elucidating TCM research (Zhang et al., 2019a; Qiu et al., 2020; Zhang et al., 2020; Du et al., 2022). The experiment workflow of metabolomics is shown in Figure 1. However, even the most comprehensive approach is unable to provide a clear upper bound on the number of metabolites. The current capacity to detect and quantify metabolites is far from comprehensive enough (Qiu et al., 2023). In mass spectrometry-based metabolomics, there are two general approaches, namely, targeted metabolomics (Liu et al., 2023a) and untargeted metabolomics (Vinayavekhin and Saghatelian, 2010). Because it can provide a methodology for the absolute quantification of identified and prospective biomarkers, targeted metabolomics is an essential component of metabolomics. However, targeted metabolomics can only be used to quantify relatively few metabolites, so there is a deficiency in overall metabolome coverage (Di Minno et al., 2021). By comparison, untargeted metabolomics can measure up to thousands of molecules at a time and can be thousands of molecules of different molecular types. Untargeted metabolomics is extremely advantageous in that it provides a large amount of data (Qiu et al., 2022).

**FIGURE 2:**
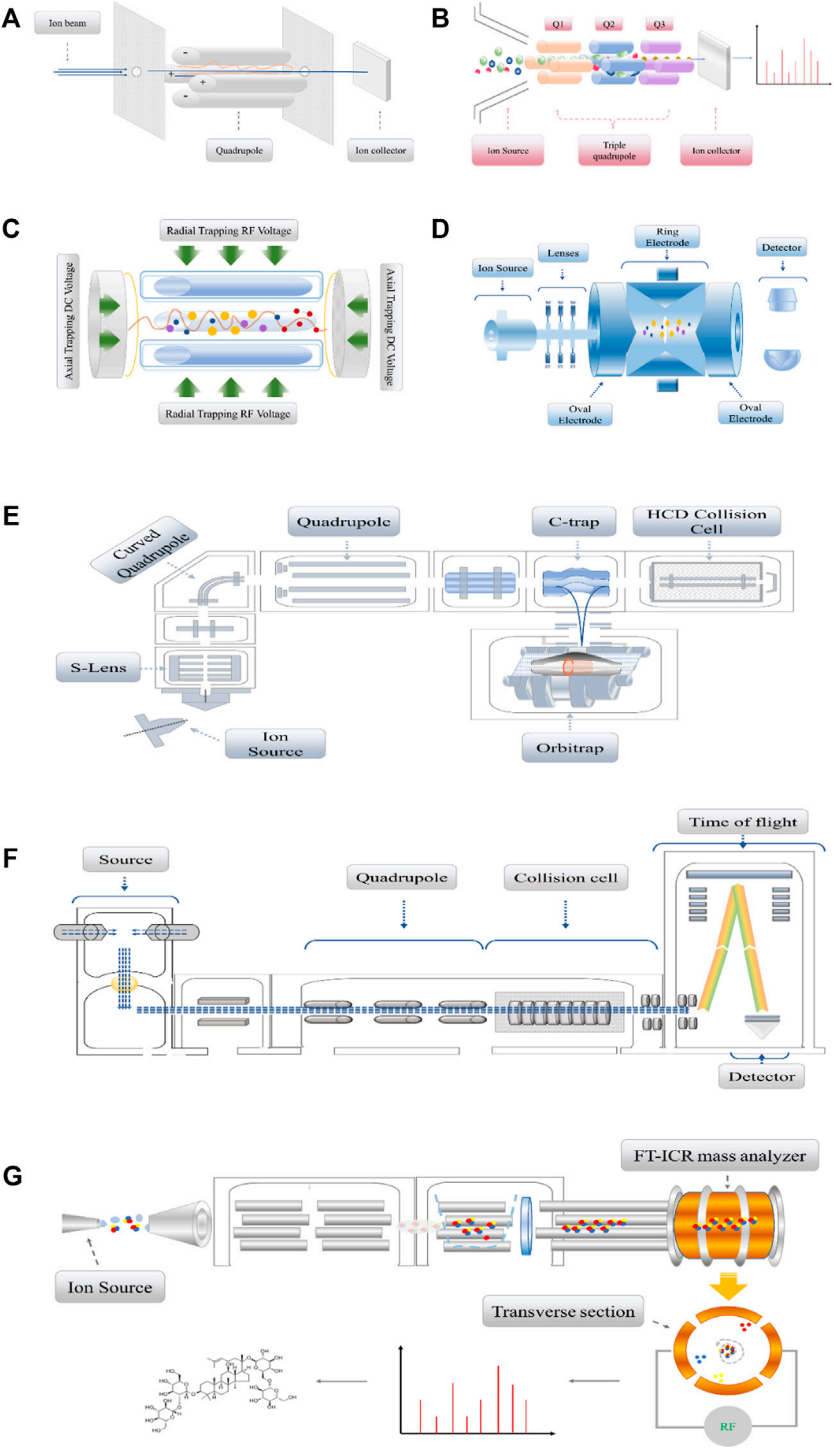
**(A)**. The single quadrupole detector consists of four parallel cylindrical or hyperbolic cylindrical electrodes which are equally spaced with the central axis to form two groups of positive and negative electrodes. The DC voltage and RF voltage in the *x* and *y* directions are applied to generate a dynamic electric field. **(B)**. Triple quadrupole detector, which by breaking the sample in the ion source to obtain specific daughter ions, daughter ions by Q1, Q2, and Q3 after the selection of the receiver into electrical signals. **(C)**. Linear ion trap, which are designed as three segments in their entirety, apply Radial Trapping RF Voltage and Axial Trapping DC Voltage between electrodes. **(D)**. 3D ion trap diagram is composed of a ring electrode and two oval electrodes. Two oval electrodes have small holes as ion channels. Generally, RF AC voltage or DC voltage is applied on the ring electrode. **(E)**. The Orbitrap detector, after ionization of the analytes in the Ion Source, will sequentially enter the Quadrupole, the C-trap, and the Orbitrap. If debris is also collected, the detected material will also be fragmented in a high-energy collision cell. It works like electrons rotate around the nucleus. **(F)**. The schematic diagram of a substance passing through the Q-TOF mass spectrometer. According to the equation of kinetic energy with mass and velocity: E = mv^2^, ions with smaller mass-to-charge ratio will obtain higher velocity, shorter flight time, and then convert into mass spectra. **(G)**. The FT-ICR mass spectrometer is a cavity with uniform superconducting magnetic field. Ions move in a circular orbit perpendicular to the magnetic field. When the cyclotron ion beam approaches a pair of traps, the image current signal will be detected on the traps, and the original data is transformed by Fourier transform to form a mass spectrum.

At present, there are four main analytical instruments to analyze and identify the structure of compounds, which are nuclear magnetic resonance (NMR) spectroscopy, infrared (IR) spectroscopy, ultraviolet-visible (UV) spectroscopy and mass spectrometry (MS). NMR spectroscopy has the characteristics of the same sensitivity to all detected substances, simple sample processing and non-invasiveness, so it can be applied in isotope labeling to trace metabolic pathways (Markley et al., 2017). However, it has the disadvantages of low sensitivity and a narrow detection range. IR spectroscopy can realize the lossless qualitative analysis of almost all organic compounds and most inorganic compounds, and has the characteristics of fast analysis speed (Putzig et al., 1994; Morris et al., 2005). UV spectroscopy is only suitable for the analysis and identification of compounds containing unsaturated bonds and aromatic ring groups, with a small scope of application, low quantitative sensitivity, and usually only reaching the microgram level (Lourenço et al., 2012). MS is suitable for the study of numerous various substances, because it has highly selective analysis, good repeatability, high sensitivity, and a wide linear range (Király et al., 2016). In the research and development of metabolomics, the most common is the combination of various chromatograms and mass spectra, such as liquid chromatography mass spectrometry (LC-MS), gas chromatography mass spectrometry (GC-MS) and Capillary electrophoresis mass spectrometry (CE-MS). The structures of the above mass spectrometers are shown in Figure 2. MS is of great help for the panoramic analysis of the metabolome. With the continuous expansion of the field of metabolomics research and the gradual maturation of MS, the application of MS technology has evolved into an essential instrument for examining the metabolomics of TCM. Depending on the characteristics of the mass analyzer, mass spectra are divided into low-resolution mass spectrum and high-resolution mass spectrum. Low-resolution mass analyzers such as quadrupole analyzer (Q), ion trap (IT), triple quadrupole (QQQ) and quadrupole linear ion trap (Q-Trap) are slightly poor in the qualitative performance of compounds, but can greatly improve the quantitative sensitivity and stability of compounds through ion selection, and the cost is relatively low, which is a reliable method for compound quantification (Mlot et al., 2011).

High-resolution mass spectrometers such as Orbitrap, time of flight (TOF) and Fourier transform ion cyclotron resonance (FT-ICR) can attain a mass resolution of nearly 10 million and are suited for correctly determining the precise molecular mass and molecular structure of substances. Although high-resolution mass spectrometry is better at quantifying chemicals, the costs of both its purchase and maintenance are substantial (Marshall and Hendrickson, 2008). The most common mass spectrometry analyzers that are based on metabolomics in the area of TCM are the analytical instruments of Q and TOF and their combinations. Besides, instruments such as Orbitrap and FT-ICR-MS were used. In this paper, the role of mass spectrometry in the metabolomics of TCM is described, and the future development of mass spectrometry in metabolomics is prospected.

## 2 Mass analyzers

### 2.1 Triple quadrupole mass spectrometer

The quadrupole mass spectrometer (QMS) is the most classical mass spectrometer (Li et al., 2018) with outstanding quantitative capabilities, and QMS accounts for the vast majority of GC-MS. QMS belongs to dynamic mass spectrometry, which does not require a magnetic field because it only uses a pure electric field to work. Consequently, the instrument’s sensitivity and resolution can indeed be altered by modifying its electrical parameters, allowing it to fulfill various analysis requirements (Ens and Standing, 2005). QMS has the advantages of straightforward structure, light weight, inexpensive maintenance, simple operation, strong quantitative ability and fast scanning speed. However, in accurate mass determination, samples are required to have relatively high purity, and the chemical background of impurities cannot exist, which can cause undetectable interference (Liu et al., 2023b), so it is less used in drug metabolomics. The most common multistage tandem MS system is the tertiary tandem mass spectrometer-QQQ. In three-stage tandem quadrupole mass spectrometry, three quadrupole analyzers are connected in series to form a QQQ sequence, which can also be called TQMS. This allows QMS to have serial functionality and gain much higher chemical specificity than single-stage, while maintaining significant quantitative power.

**FIGURE 3:**
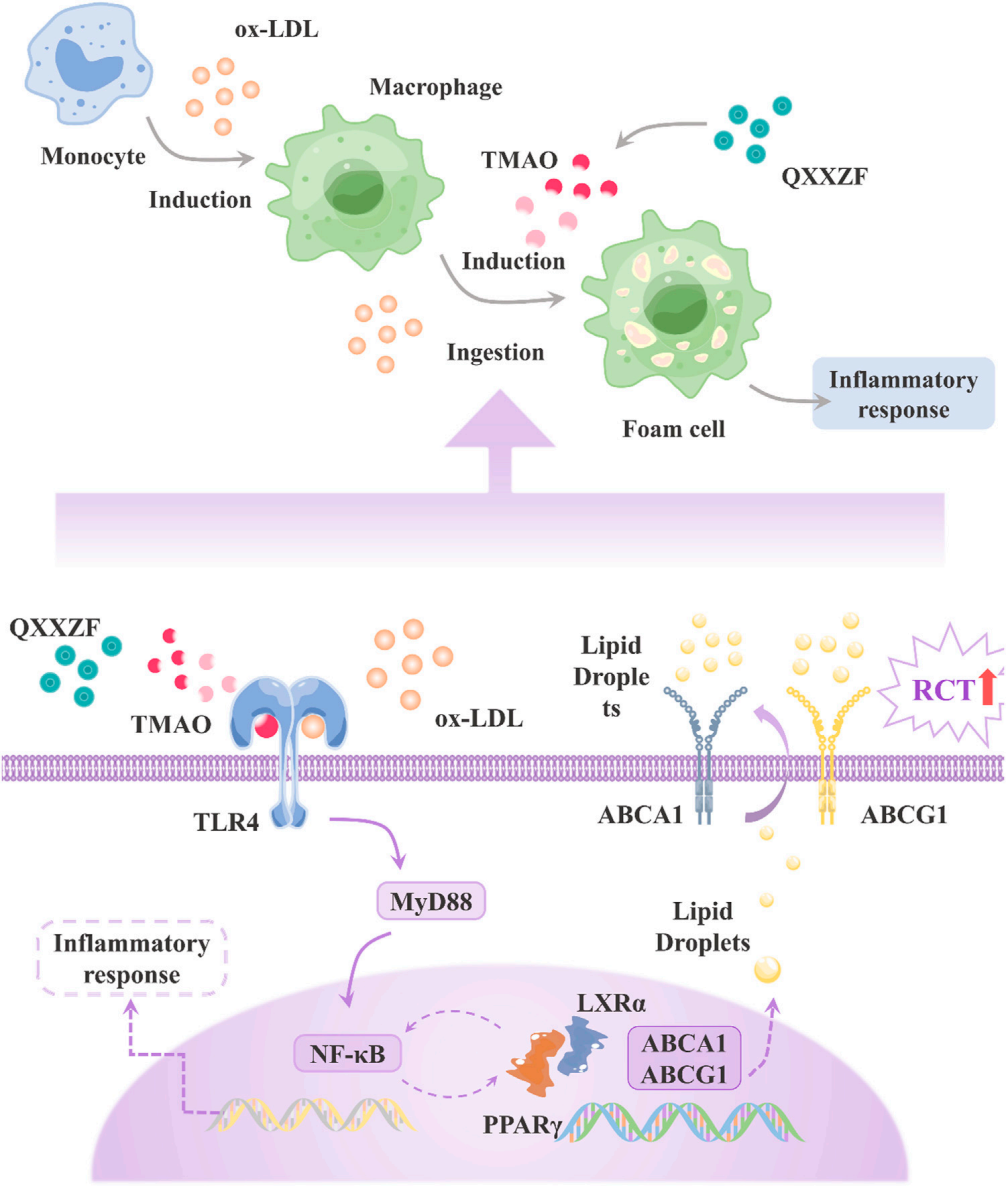
Mechanism of Qing-Xue-Xiao-Zhi formula in attenuating atherosclerosis. QXXZF, Qing-Xue-Xiao-Zhi formula; ox-LDL, oxidized low-density lipoprotein; TMAO, trimethylamine N-oxide; RCT, reverse cholesterol transport; ABCA1, ATP-binding cassette A1; ABCG1, ATP-binding cassette G1; PPARγ, peroxisome proliferator-activated receptor γ; LXRα, liver X receptor α; TLR4, Toll-like receptor 4; MyD88, Myeloid Differentiation primary response 88; NF-κB, Nuclear Factor kappa B.

The most common multistage tandem MS system is a three-stage tandem mass spectrometer--QQQ. In three-level tandem quadrupole mass spectrometry, three quadrupole analyzers are connected in series to form a QQQ sequence, which can also be called TQMS (Yost and Enke, 1978). This allows QMS to have serial functionality and gain much higher chemical specificity than single-stage, while maintaining significant quantitative power. The TQMS workflow diagram is shown in Figure 3. In this way, the qualitative ability of mass spectrometry is strengthened, and it is widely utilized as a confirmatory detection approach for QMS in the detection standards. TQMS includes a wide range of capabilities beyond the standard product ion scanning operation, including SRM, MRM, precursor scan, neutral loss, and others (Rubino, 2020). Therefore, it is particularly suitable for combination with LC and is now widely used in metabolomics for the screening and identification of metabolites and the metabolic transformation of active components of TCM.

### 2.2 Quadrupole time-of-flight tandem mass spectrometer

TOF-MS performs well in terms of fast scanning capability, wide mass range, and high resolution (Liang et al., 2015). Q-TOF combines the stability of QSM with TOF functionality by using QMS as a quality filter and TOF-MS as a mass analyzer for coupling. The most common arrangement involves three independent quadrupoles executing the sequential tasks of collision-induced dissociation, parent ion selection, and collision cooling. Then the TOF spectrometer is filled with orthogonal ions for the purpose of obtaining m/z measurements (Ens and Standing, 2005). Q-TOF offers superior resolution spectra as well as MS/MS experiments. At the same time, it has the characteristics of a fast running speed, which creates the conditions for providing comparable sensitivity and a shorter method development time. Q-TOF-MS coupled to liquid chromatography is one of the most commonly used instruments in metabolomics research. In spite of its moderate mass resolution and mass precision, it can be compensated by the very fast scan rate, thus better defining the chromatographic peak (Zhang et al., 2014). However, the flight tube vacuum cavity of Q-TOF MS is bulky and has poor quality stability, and most instruments require real-time correction. It has advantages over QQQ configuration in high performance MS/MS measurements of biomolecules (Zhang et al., 2013b). TOF/MS also has the advantages of a wide mass analysis range and a large data set, making it particularly suitable for use in tandem analysis with ultra-high performance liquid chromatography and tandem analysis with quadrupole mass spectrometry, which can obtain secondary mass spectra of low-abundance chemical components and is suitable for the analysis of complex TCM system components. However, because Q-TOF MS lacks a multi-stage mass spectrometry function, it must be combined with other tandem mass spectrometry when inferring or confirming the structure of TCM components. In the experiment, except for the identification and analysis of TCM components using UHPLC-Q/TOF-MS alone, some components will also be quantified and further elucidated by Q/TOF-MS combined with TQ-MS (Liu et al., 2018a; Lin et al., 2020). At the same time, the *in vivo* metabolites of TCM can also be identified and characterized by UPLC-QTOF/MS (Mi et al., 2019).

### 2.3 Ion trap mass spectrometry

IT-MS is a three-dimensional quadrupole field composed of a toroidal electrode with a hyperboloid section and a pair of upper and lower end electrodes (Elschner, 1953). The principle of IT is to store ions in the trap, and then change the electric field to push ions out of the trap according to different mass-to-charge ratios for detection. Due to their uncomplicated construction, compact size, outstanding sensitivity, and relatively low vacuum demands, IT mass analyzers have emerged as the preferred choice for the creation of miniature mass spectrometers (Snyder et al., 2016). Two-dimensional linear ion trap (LIT), which is an improved form of conventional 3D ion trap, is not constrained by radiofrequency potential in the axial direction as is the case with 3D ion trap (Li et al., 2021a). TCM metabolites are emerging as important sources for the discovery of pharmacologically active substances and new drugs. However, the large internal destruction and small amounts of metabolites in biological matrices make it challenging for TCM to identify and discriminate between *in vivo* components. The advent of IT mass spectrometry has provided solutions to this challenge. However, the disadvantage of IT is that it has a fair resolution and cannot give accurate molecular weight information. Optimization is therefore achieved by coupling with other types of mass analyzers. The quadrupole ion trap (Q-Trap) that adds auxiliary radiofrequency to the QQQ can do selective excitation and provide the functional advantage of multistage serial for the QQQ (Douglas et al., 2005). Ion trap-time-of-flight (IT-TOF-MS) mass spectrometry uses a 3D ion trap as a mass selector and reactor, which combines the multi-stage mass spectrometry ability of the IT with the excellent resolution ability of TOF-MS (Liu, 2012). Linear ion trap-time-of-flight (LIT-TOF-MS) mass spectrometry uses a LIT as a mass selector and reactor, which combines the highly sensitive multistage serial capacity of the LIT with the high-resolution capacity of TOF mass spectrometry (Kosma et al., 2021). Combining the information of IT and TOF mass spectra for structure identification can improve the accuracy of the results and is a common strategy for the identification of TCM components. The elemental composition of the components was deduced by determining the exact molecular weight of TOF. The fragmentation information was then obtained using the multi-stage mass spectrometry function of IT to confirm the structure. LIT-Orbitrap mass spectrometers have the ability to screen and confirm compounds with multiple residues in complex substances (Kosma et al., 2021). LIT-Orbitrap has the dual confirmation function and precise molecular weight of MSn, which can explain the molecular cleavage process, and is also an important tool in the field of metabonomics of TCM.

### 2.4 Orbitrap

The Orbitrap, the first high-performance mass spectrometer introduced in early 2000, which uses electrostatic fields to capture ions, is another mass analyzer of the Fourier transform series and can usually be used in combination with other mass analyzers (Zubarev and Makarov, 2013). Two exterior electrodes and an electrode in the center of the Orbitrap mass spectrometer serve as both an analyzer and a detector. Ions enter the Orbitrap and are trapped by electrokinetic extrusion, then they oscillate around at the center electrode and among the two external electrodes. When ions are tangentially introduced into the logarithmic DC field between these two electrodes, they begin to oscillate radially around the lead and are eventually ejected at the end of the trap. Different ions oscillate at different frequencies and thus get separated. By determining the oscillation frequency that the ion generates on the external electrode, image flow monitoring can be utilized to acquire the mass spectrum of an ion (Hu et al., 2005; Perry et al., 2008; Makarov and Scigelova, 2010). The first Orbitrap mass analyzer was developed to build a new spectrometer avoiding the disadvantages of existing instruments, such as the complexity and size of the FT-ICR analyzer; the limitations of resolution, dynamic range, and sensitivity of the TOF analyzer; and the limited mass accuracy of the IT analyzer (Eliuk and Makarov, 2015). Orbitrap closes the gap between TOF and FT-ICR quality analyzers. And this instrument is currently more suitable for targeted analysis. However, Orbitrap-MS has a lower acquisition rate than Q-TOF MS, and its resolution interacts with the scan rate, and the resolution decreases significantly at high scan rates. It is difficult to obtain the secondary spectra of low-abundance chemical constituents in TCM under the traditional data-dependent scanning mode (DDA). Therefore, this instrument is currently more suitable for non-target analysis and target analysis of active components of TCM. The Orbitrap-MS, in combination with the LC, provides approximately 500,000 resolution capabilities (Pan et al., 2020; Stettin et al., 2020). In contrast, since its development, Orbitrap-MS integrated with GC has had few applications in the field of metabolomics. It is possible that LC separating has been shown to offer the optimum balance between uncomplicated sample processing and metabolome coverage, making it the preferred method for MS-based metabolomics.

### 2.5 Fourier transform ion cyclotron resonance mass spectrometer

Ion cyclotron resonance spectrometry (ICR) and contemporary digital technology were combined to create Fourier transform mass spectrometry (FT-MS), also known as Fourier transform ion cyclotron resonance mass spectrometry (FTICR-MS). FTICR-MS has the highest resolving power and is often used as equipment for high-end scientific research, and its unique analytical characteristics make it an essential tool for studies on proteomics, metabolomics and complex mixtures. A common method for micromolecule and proteomic analysis uses FTICR-MS in conjunction with ion preselection and fragmentation equipment and its bonding with reversed-phase LC. The high fidelity of detection linearity and frequency determination are inherent properties of FTICR-MS, which can realize the resolution, mass accuracy and dynamic range (Scigelova et al., 2011; Nikolaev et al., 2016). Since its invention, the fundamental principle of FT-ICR-MS has remained the same: Internally or externally generated ions are radially captured inside the strong electromagnetic field in conjunction with the poor axial electromagnetic current, and image currents are observed from connectedly energized captured ions, culminating in time-domain signals that are imaged and transformed into frequency-domain spectra using FT before being converted into mass spectra. Additionally, cooling circulation is provided for the cryogenic preamplifier within the FT-ICR, increasing sensitivity (Comisarow and Marshall, 1974; Nicolardi et al., 2015; Choi et al., 2012; Qi and O’connor, 2014). FT-ICR-MS has a strong resolving power and can be used in combination with different ionization sources to detect compounds with different polarities. Its ultra-high resolution and mass accuracy can provide accurate elemental composition of compounds, giving it significant potential and advantages in the composition and structure of unknowns, as well as the rapid analysis of complex mixtures. However, the comprehensive grasp of the data machining methods used tends to lag behind that of the instrument, and the majority of data machining algorithms used in FT-ICR have not been thoroughly studied; as a result, expert skills and experience in FT-ICR procedure and data analysis are still essential to achieving the high performance of FT-ICR.

### 2.6 Ion mobility mass spectrometer

In the traditional MS, the new separation and measurement factor of ion mobility is added, thus constituting the IM-MS system. IM-MS has multidimensional separation, which increases peak capacity, shortens analysis time, separates structural analogues or isomers, and has multiple fragmentation modes and acquisition modes. Experimental data that cannot be provided from MS alone when IM is coupled with MS. Ion-size isomers can be measured by adding an ion migration cell to the MS. Isobaric lines and conformational isomers are separated, and chemical noise is decreased, which are characteristics of IMS. Additionally, ions with a similar charge state and those with structural similarities can be grouped into categories according to the lines where their mass-mobility relationships appear (Kanu et al., 2008). At present, there are four ion mobility separation methods used with MS, including drift time ion mobility spectrometry (DTIMS) (Latif et al., 2021), traveling wave ion mobility spectrometry (TWIMS) (Shliaha et al., 2014), differential migration spectrometry (DMS), also known as field asymmetric waveform ion mobility spectrometry (FAIMS) (Costanzo et al., 2017) and aspiration ion mobility spectrometry (AIMS) (Ratiu et al., 2017). Especially for compounds that are not easily separated chromatographically, IM-MS has great separation advantages. Combined with high resolution mass spectrometry, it has significant advantages in the study of the material basis of TCM. IM has been linked to additional mass spectrometers such as QQQ, IT, and Orbitrap in addition to TOF, which is typically the main mass instrument associated with IM-MS. IM-MS can be readily combined with other cutting-edge technologies, such as capillary electrophoresis and supercritical fluid chromatography. The characteristic parameter ion mean collision cross-sectional area (CCS) determined by IM-MS is an inherent property of compound ions and has significant advantages in compound characterization. However, the CCS database of chemical components of TCM is still blank, and the construction of the CCS database of chemical components of TCM is of great significance for the analysis and research of TCM. The advantages and disadvantages of the above mass spectrometers and their scope of application in the field of TCM research are shown in Supplementary Table S1.

## 3 Application

### 3.1 Pharmacodynamic evaluation

Effectiveness is a fundamental property of a drug. Despite the fact that TCM is frequently used in clinical settings, it is still challenging to demonstrate its usefulness through science (Cao et al., 2015; Ma et al., 2016). Further consolidating and improving the efficacy of TCM and greatly enhancing its contribution to medical and health security in China has become a key issue for the sustainable development of TCM. TCM effectiveness evaluation is a necessary step in determining TCM quality markers and pharmacodynamic material basis. However, the clinical effectiveness of TCM is often related to biochemical indicators; in addition, mass spectrometry should be used to prove that the effectiveness of TCM is directly related to clinical indicators from a molecular point of view and at a microscopic level. Combining *in vivo* and *in vitro* studies, the effectiveness of TCM was scientifically expressed, and the overall pharmacodynamic action process of TCM was efficiently analyzed under the premise of effectiveness. Tang et al. (2020) used UPLC-Q-TOT-MS to quantitatively analyze the multiple biotransformation products of Xian-Ling-Gu-Bao (XLGB) with rat intestinal bacteria, and this study method successfully described the dynamic contour of thirty-one biotransformation substances of XLGB. He et al. (2022) studied the role of Jigucao capsule (JGCC) in the treatment of Dampness-heat Jaundice syndrome (DHJS) using a classical strategy, which is a research technique of UPLC-QTOF-MS united with pattern identification along with metabolomics applications and digital databases JGCC has been found to call back twenty-five potential biomarkers, including arachidonic acid, L-urobilin, etc. Yao et al. (2022) used UPLC-QTOF-MS to find that Longzuantongbi granules effectively regulated 11 biomarkers associated with lipid metabolism and amino acid metabolism, such as lysophosphatidylcholine, alpha-3-hydroxybutyric acid, alpha-linolenic acid, arachidonic acid, and 12-HETE. Wang et al. (2021) demonstrated that Liu Shen capsule treats respiratory diseases by altering airway microbiota, such as Bacteroidetes and Proteobacteria, based on UPLC-QTOF-MS. Yang et al. (2022) explored the antidiabetic effect of red ginseng on type Ⅱ diabetes with a focus on UPLC-Q Exactive-MS Technology and found that disturbances in regulatory pathways such as D-arginine and D-ornithine metabolic activities, tryptophan bioconversion, taurine, and hypotaurine metabolism were improved after red ginseng intervention. Liu et al. (2022) used UPLC-IT-TOF-MS/MS to qualitatively analyze the metabolites of berberine in human hepatocellular carcinoma cells to elucidate the biodistribution and pharmacokinetic profile of berberine and its metabolites in hepatocytes. Zhao et al. (2022) targeted analysis of 29 kinds of energy metabolites in myocardial tissue based on UPLC-QQ-MS, and verified that Naoxintong Capsules played a role in treating myocardial infarction by affecting part of energy metabolism.

### 3.2 Quality markers and quality control

In the clinical setting, the effectiveness of TCM is crucial. Contrary to modern medicine, TCM’s multiple active ingredients are affected by a variety of parameters, including the source, provenance, and processing methods of medicinal ingredients (Bai et al., 2018). The non-systematic nature of TCM quality control also seriously affects its efficacy and credibility. The idea of quality-marker (Q-marker), according to TCM features was proposed by Professor Liu ChangXiao, providing new research opportunities for TCM quality management (Liu et al., 2018b; Wu et al., 2018). Q-markers of TCM are substances that are naturally occurring in medicinal materials and components, such as processed goods, extractions, and formulations, or that are created during manufacturing and preparation. The Q-marker approach essentially emphasizes the core meaning of TCM’s quality qualities, which not only indicate security but also represent the effectiveness of treatment and represent a fundamental advance in standard evaluation models and ideas (Yang et al., 2017; Ren et al., 2020). To investigate the potential Q-marker of the Periplocae Cortex, UPLC/Q-TOF MS and network pharmacology approaches were integrated. A total of nine components, such as periplocin, periplogenin, periplocymarin, etc. were selected as Q-markers for the Periplocae Cortex (Li et al., 2021b). Li et al. (2022) analyzed the Q-markers of Wutou decoction (WTD) according to Pearson correlation analysis and UPLC-Q/TOF-MS and identified 12 compounds, including aconitine, ephedrine, quercetin, astragaloside IV, paeoniflorin, and glycyrrhizic acid, etc. as Q-markers of WTD. Zhang et al. (2015) used Carbonized Typhae Pollen (CTP) as an example to establish a discovery strategy for Q-markers of carbonized TCM. Using UPLC-QTOF-MS, six Q-markers of CTP were found to include kaempferol-3-O-neohesperidoside, isorhamnetin-3-O-neohesperidoside, sorhamnetin, naringenin, quercetin, isorhamnetin-3-O-rutinoside and isorhamnetin. Liang et al. (2021) identified eight compounds, including emodin, chrysophanol, magnolol, hesperidin, geniposide, etc. as potential Q-markers for Chaiqin chengqi decoction (CQCQD) based on UPLC-QQQ-MS. Chu et al. (2022) found sweroside, paeoniflorin, liquiritigenin, chlorogenic acid, calycosin-7-glucoside, formononetin and 3-butylephthalide to be Q-markers of the Mailuoshutong pill based on TCM theory and metabolomics techniques. Wang et al. (2023) used UPLC-Q-Exactive Orbitrap MS to identify five active ingredients in Bushen Huoxue Prescription as potential Q markers against diabetic retinopathy, tanshinone IIA, puerarin, ajugol, protocatechuic acid and panaxatriol. Li et al. (2023) used UHPLC-LTQ-Orbitrap MS to analyze and identify the active components in Panax notoginseng and found a new efficacy grading marker alkynol related to Panax notoginseng, which can be used as a new grading quality marker.

The quality marker of TCM is the basis of a modern TCM research system that combines macroscopical and microscopic aspects and includes the study of compatibility law of TCM compounds, the basic study of chemicals and the modern pharmacological study from the molecular point of view. The determination of TCM quality markers is based on the overall view of “pharmacodynamic components,”, that is, those that have clear chemical structure and biological activity. It is conducive to carrying out layer-by-layer analysis of the pharmacodynamic components that play an important role in TCM, and gradually elucidating its pharmacological effects and mechanism of action, which is the premise for traditional Chinese medicine to carry out subsequent research.

### 3.3 Formula compatibility

TCM formula has always been the most common type of medication in clinical research, and it is a group of drugs that are properly compatible according to the composition principle after selecting the appropriate dosage of drugs after determining the treatment method based on syndrome differentiation examination. TCM formulas containing two or more medicinal herbs tend to obtain better efficacy and fewer side effects than the single herbs (Zhou et al., 2017). Jun-Chen-Zuo-Shi in Chinese (king, minister, assistant and guide) is one of the most typical and significant theories, which vividly defines the diverse roles of various components in TCM (Luan et al., 2020). Herb pairings were crucial to the progress of TCM, and their appearance expanded its range of applications and established the organizing tenets of formulas. The compatibility principles of TCM formulae include considering herbal properties (hot, cold, warm, and cool), herbal taste (pungent, sweet, bitter, acidic, and salty), and pharmacodynamic trends (entering meridians, going up and down, and floating, etc.), as in modern medicine: Synergism and attenuation (Wang S. et al., 2012; Zhang et al., 2015).

Gu et al. (2021) investigated the synergistic effect of Jinlingzi San (JLZS) on Fructus Toosendan (FT) and Rhizoma Corydalis (RC) based on UPLC-QQQ-MS. Synergistic effects of the combination of FT and RC were observed at the pharmacokinetic level, slowing the clearance of tertiary alkaloids and improving their intake and bioavailability to some extent. Sun et al. (2021b) used UFLC-QTRAP-MS targeted metabolomics to elucidate the contribution of Salvia miltiorrhiza and Pueraria lobata in the treatment of acute myocardial ischemia (AMI) in Xin-Ke-Shu and found that the regulatory effect of cardioprotection during AMI was significantly lost in the deficient group lacking Salvia miltiorrhiza and Pueraria lobata, especially the mediation of FFA metabolism. Zhou et al. (2021) investigated the compatibility principle and salt treatment of salt-processed Foeniculi Fructus& Salt-processed Psoraleae Fructus (sFF&sPF) by UHPLC-QTOF-MS. The results showed that sFF&sPF group had the best efficacy in the treatment of diarrhea and polyuria, and salt treatment would briefly affect the correlation between pharmacodynamic components and endogenous metabolites. Chen et al. (2021) used pharmacokinetics and metabolomics, relying on UHPLC-Q Exactive Orbitrap-MS technology, to elucidate the compatibility mechanism of Radix Bupleuri-Radix Paeoniae Alba (RB-RPA). The results showed that the RB-RPA combination could dramatically enhance the bioavailability of 11 drug ingredients and raise neuroprotective and anti-inflammatory activity. Guo et al. (2023) used LC-IT-TOF-MS to compare the component differences between single herb and Zhi-Zi-Chi decoction (ZZCD) compounds, while the solubility of toxic substances and active components was found to affect the attenuation and synergy of the compound by quantitative characteristic compounds, laying the foundation for optimizing the compatibility of ZZCD. Dong et al. (2023) used UFLC—QTRAP-MS to compare the pharmacokinetic differences between Sijunzi decoction and “quewei” Sijunzi compound (one medicinal herb was randomly removed) and found that the compatibility of the four herbs could change the pharmacokinetic properties of the compound and justify oral administration of Sijunzi decoction.

The research on the chemical constituents of Chinese herbal compounds alone is not enough to clarify the compatibility law of Chinese herbal compounds and their basic efficacy and mechanism of action. As a result, using mass spectrometry, it is necessary to analyze the qualitative or quantitative changes in active ingredients of drugs before and after compatibility, study the pharmacokinetic interactions *in vivo*, that is, the effects of TCM compatibility on *in vivo* processes such as absorption, distribution, metabolism, and excretion, and finally reflect the synergistic or resistant biological effects in TCM compounds through effect indicators, bioavailability, and target recognition.

### 3.4 Mechanism of action and target exploration

Although there is only one drug, single herbs contain a variety of active ingredients. While TCM formulas are composed of several herbs and dozens of components, these components are decomposed into countless chemical molecules, so elucidating the mechanism of action of TCM is a very difficult thing (Hao et al., 2017). Metabolomics based on mass spectrometry combined with multivariate statistical analysis can analyze endogenous metabolites, study their species, quantities, and changes under the action of internal and external factors, and perform group index analysis through systematic integration to reflect the dynamic changes of endogenous metabolites in organisms. Li et al. (2021c) studied the mechanism of action of the Qing-Xue-Xiao-Zhi formula (QXXZF) in the treatment of atherosclerosis based on the serum metabolomics of UPLC-Q-TOF-MS. This paper has found that QXXZF can inhibit the development of atherosclerosis by reducing atherosclerotic plaques in the aorta and aortic root, reducing the content of oxidized low-density lipoprotein and trimethylamine N-oxide in serum. Moreover, QXXZF can also promote reverse cholesterol transport by regulating the expression of related genes (PPARγ/LXRα/ABCA1/ABCG1). Wang et al. (2022a) explored the mechanism of action of Qing-Xin-Jie-Yu Granule (QXJYG) in the treatment of atherosclerosis by LC-QTOF-MS. Finally, QXJYG was found to reduce mRNA levels of IL-6 and IL-1β in the aorta while remodeling gut microbiota and associated bile acid levels. Zhang et al. (2022c) investigated the mechanism of Sheng Mai San (SMS) in the treatment of liver injury by UHPLC-QTRAP-MS combined with the pharmacodynamics strategy. The results showed that SMS reconstructs mitochondria to achieve therapeutic effects by altering AST/ALT ratio, regulating glycolysis and TCA cycle. Luo et al. (2022) found that emodin ameliorates ulcerative colitis by modulating the PPARγ signaling pathway. Wang et al. (2022b) evaluated the metabolites of honeysuckle by UPLC-QTOF-MS isolation and found that chlorogenic acid and swertiamarin could regulate the main mediators of inflammation, which in turn affected the phosphatidylinositol-3-kinase-AKT and MAPK pathways. Taking QXXZF as an example, its mechanism of action is shown in Figure 3.

**FIGURE 4:**
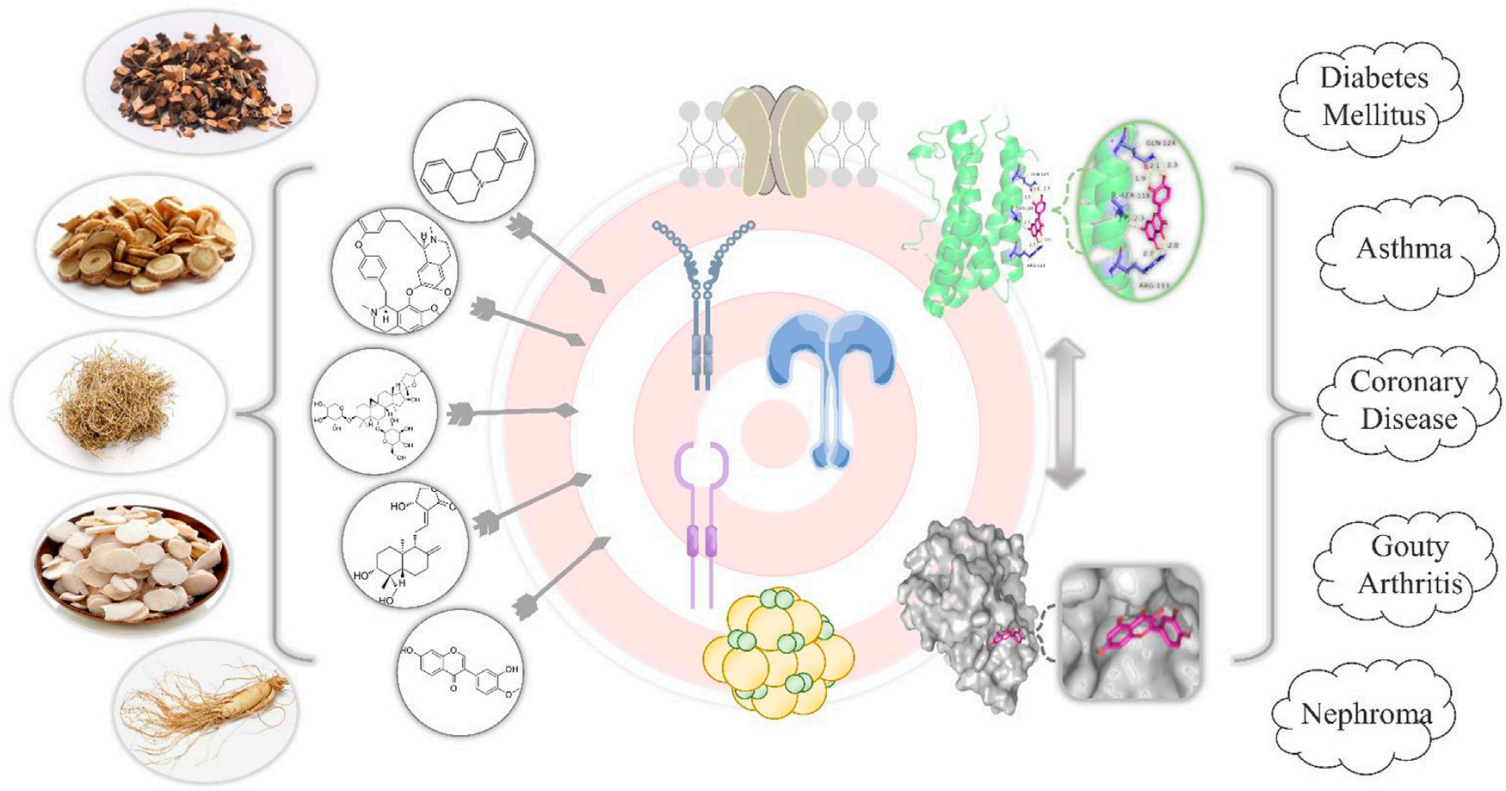
‘Drug-Ingredient-Target-Disease’ Network Diagram. Drug ingredients bind to common targets such as enzymes, ion channels, receptors, or other biomolecules. In this figure, quercetin is used as an example to show its docking diagram with the stick structure and surface structure of IL6 target.

**FIGURE 5:**
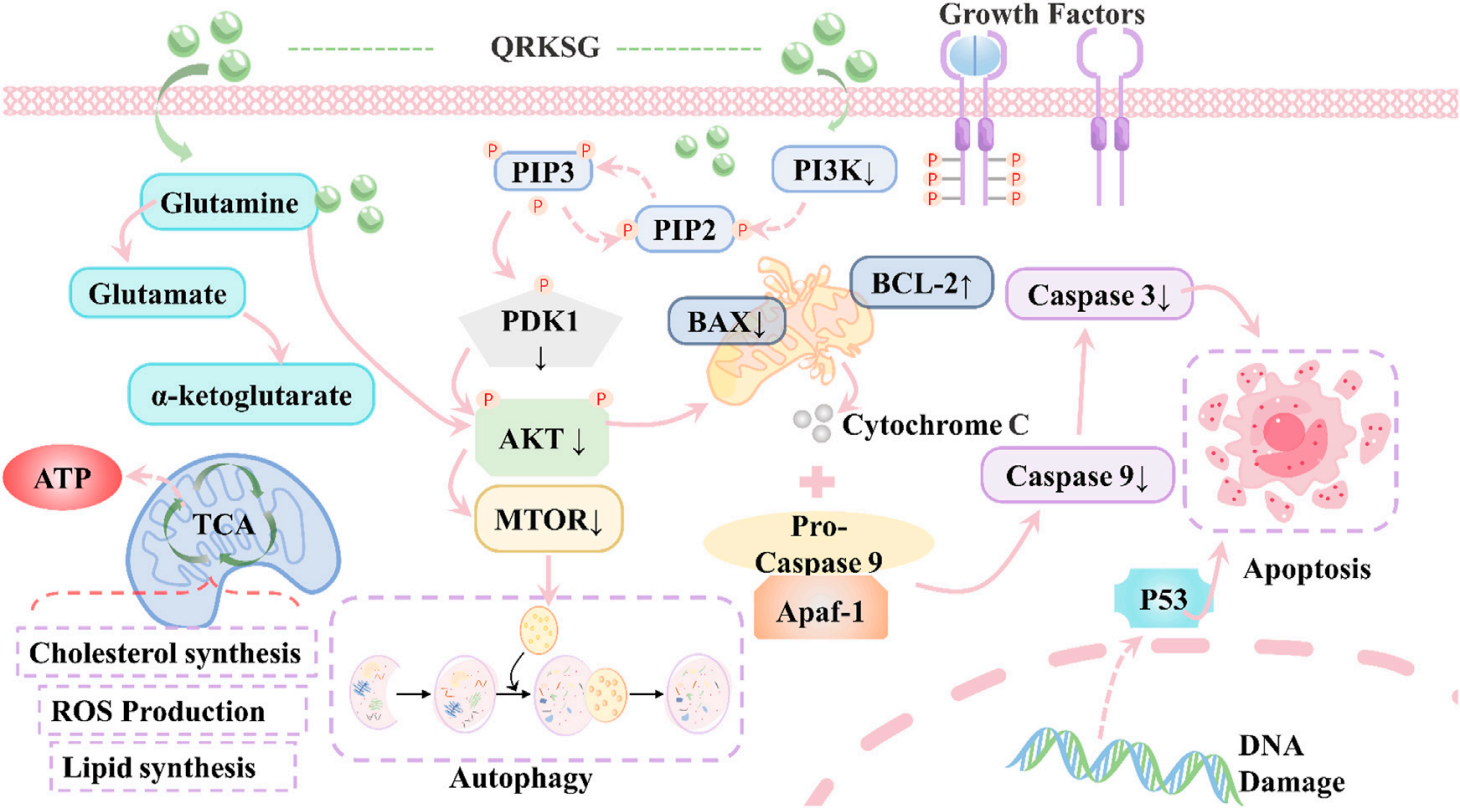
Mechanism of Qing-Re-Ka-Sen Granule in Treating Nephrotic Syndrome. QRKSG, Qing-Re-Ka-Sen granule; PI3K, Phosphatidylinositol3-kinase; PIP2, Phosphoinositide 2 kinase; PIP3, Phosphoinositide 3 kinase; PDK1, Pyruvate dehydrogenase kinase 1; AKT, AKT Serine/Threonine Kinase; mTOR, mammalian target of rapamycin; BAX, BCL2-associated X protein; BCL2, Apoptosis Regulator; TCA, tricarboxylic acid cycle; ROS, reactive oxygen species; P53, tumor suppressor gene.

Many studies showed that the main active components of TCM can target one or more specific molecules and thus perform a variety of clinic treatment functions (Shen and Yin, 2021). Exploring TCM from the methodical sentiment and the molecular criterion changes the research paradigm from the previous “one-target and one-drug” model to a novel “multiple targets and multiple constituents” model. Based on the exploration of the drug-disease relationship in the comprehensive pharmacology of TCM, the network of “drug active ingredient-target-disease” was established and enriched, as shown in Figure 4, which was used to analyze the functions of key target genes, metabolic pathways and their “cores”. Construct a comprehensive network framework for TCM-target interactions. Based on the biological effect factors and the regulation mechanism of TCM on the targets and pathways of disease, strengthen the precise positioning and provide accurate treatment of TCM (Li and Zhang, 2013; Wang et al., 2018; Wei et al., 2021). Duan et al. (2021) studied the mechanism of Qing-Re-Ka-Sen granule (QRKSG) in the remedy for nephrotic syndrome (NS) *via* integrating UPLC-QTRAP-MS, GC-MS, Western blotting and molecular docking techniques. Ultimately, it was discovered that the main targets of the QRKSG for the therapy of NS were AKT1, MTOR, CASP3, and BCL2L1. Guo et al. (2022) found that Qifenggubiao granules achieved immunomodulatory effects by acting on targets such as HSP90AA1, PPARA, PTGS2, CASP3, AKT1, IL6, MAPK3, MAPK1, ESR1, and PPARG. Figure 5 illustrates the target action for QRKSG. Li et al. (2021d) used data mining technology combined with HPLC-Q-Exactive MS/MS to comprehensively analyze the key targets of *Hydroxysaffloryellow* A in the treatment of traumatic brain injury, as PTGS2, XDH, NOS1 and ACHE. Huang et al. (2022); Zhang et al. (2022d) used UPLC-QTOF-MS to discover potential targets of Chaigui Granule in the treatment of depression and key targets of Rheum officinale Baill. in the treatment of thrombotic diseases, respectively. Wang et al. (2020c) characterized 20 compounds in Zi-shen pill ultrafiltrate using FT-ICR MS and found four active ingredients that inhibit 5-lipoxygenase to treat benign prostatic hyperplasia.

### 3.5 Pharmacophoric substance discovery

A large number of research studies have shown the utility of metabolomics in discovering the pharmacodynamic material basis of TCM and TCM formulations (Han et al., 2020). The pharmacodynamic material basis of TCM refers to the general term for the chemical components contained in TCM that can express the clinical efficacy of drugs. All the effective components in the Chinese herbal formula were separated and identified by mass spectrometry, and the changes in endogenous metabolites after the compound acted on specific diseases were further studied to elucidate the relevant metabolic pathways. Finally, the pharmacodynamic components that could prevent and treat specific diseases were selected. To find the active constituents of Xiaoxuming decoction (XXMD) in the therapy of ischemic epilepsy, Luo et al. (2019) used UPLC/Q-TOF MS in conjunction with fast separation liquid chromatography-triple quadrupole linear ion trap mass spectrometry (RRLC-QTRAP MS(n)). 48 different substances were found in XXMD by qualitative examination; 33 of these substances underwent quantitative study, and the most prevalent substances were found to be monoterpenes, flavonoids, and cyanophoric glycosides. Huang et al. (2021) compared the metabolic profiles of Uncaria rhynchophylla. (Miq.) Miq. ex Hvail. and Uncaria hirsute Havil. based on UHPLC/Q-Orbitrap-MS. In this study, six potential vasodilator compounds with relaxant effects on mesenteric arteries were selected using multivariate statistical analysis, including corynoxeine, isocorynoxeine, isorhynchophylline, rhynchophylline, and hirudin. Sun et al. (2022) identified glycyrrhizic acid, kaempferol, cinnamaldehyde, catechin daidzein, and caffeic acid as the primary therapeutic efficacy components of Lingguizhugan decoction in the treatment of heart failure using UHPLC-Q-TOF-MS/MS in conjunction with cell experiments and digital storage mining; Kong et al. (2022) used UPLC-QTOF-MS to explore the core active ingredients of Keluoxin (KLX) in the treatment of diabetic retinopathy. The results showed that rhein, astragaloside IV, emodin, chrysophanol and other compounds may be the pharmacodynamic basis of KLX. Liao et al. (2023) isolated and identified the specific binding components of Huanglian Jiedu Decoction to human umbilical vein endothelial cells by a combination of UPLC-Orbitrap-MS and found 13 active components such as geniposide, thapsigin, baicalin, and berberine as pharmacodynamic components that promote angiogenesis. Gao et al. (2023) isolated and identified 45 compounds from the leaves of Amomumvillosum Lour., with the help of HPLC-QTOF-MS technology and further experimented that 14 compounds (one unknown compound and thirteen known compounds) could prevent and treat diseases caused by inflammation and oxidative stress. Liu et al. (2023b) analyzed the components of Danshen decoction by UHPLC-Q-Orbitrap-MS technology, combined with network pharmacology, to elucidate that 35 compounds in its active components are potential pharmacodynamic materials for the treatment of cardiovascular diseases.

## 4 Future perspectives and conclusion

Metabolomics, as a science that can comprehensively analyze the metabolome, can elucidate the mechanism of action and the change rule of action of TCM by specifically comparing the changes in metabolic profile. It provides a powerful grasp of “multi-component-multi-target” for TCM, and provides a more scientific and reasonable comprehensive explanation for elucidating TCM. As the most important instrument for the analysis and identification of compound structure, MS lays the foundation for the scientific elaboration of the metabolome change contour, pharmacodynamic material basis, mechanism of action, safety, and compatibility principles of TCM. Metabolomics also emerged as a result of the development of MS. With the addition of high-tech and more MS databases, the characterization and identification of metabolites are also more accurate.

With the advancement of science and technology and the passage of time, the types of MS and MS-based analysis technology have been continuously updated, reformed and broken through, and the sensitivity, scope of application, throughput and identification technology of instruments have been greatly improved. The development of MS has contributed to the promotion of metabolomics applications, giving researchers a more comprehensive understanding of small molecule metabolites. Although mass spectrometry has the traits of high resolution, good precision, and maximum throughput, the research progress of metabolomics is still slow due to the complexity of organisms, the huge number of metabolites, and unrepeatable experimental protocols in different laboratories. Different types of MS have their own advantages, and appropriate mass analyzers are selected according to different sample characteristics and requirements. Compared with the acquisition of data, the subsequent processing and mining of data are the bottleneck problems facing metabolomics. Although the existing metabolome database has shortcomings in terms of scale, the open MS database and shared metabolome dataset provide convenience for the qualitative identification of metabolomics. Introducing the quick algorithms of computers into the field of metabolomics is of great help to the annotation as well as the accuracy of the data. The research direction of multi-omics conforms to the root of the overall regulation of organisms, and multidisciplinary cross-fusion can promote the development of metabolomics.
