# Supplementary material for: Mass spectrometry-based metabolomics for discovering active ingredients and exploring action mechanism of herbal medicine
Source: Front Chem. 2023 Mar 31;11:1142287. doi: 10.3389/fchem.2023.1142287 (PMC10102349; doi:10.3389/fchem.2023.1142287)
Supplement: Supplementary file 1 [file Table1.docx]

Table 1 Comparison of advantages and disadvantages of commonly used mass spectrometry

| Type | Applied Range | Advantages | Disadvantages | Examples |
| --- | --- | --- | --- | --- |
| QQQ | Quantitative analysis of known compounds;  Metabolic transformation of active Ingredients of TCM;  Detection of potential toxic ingredients of TCM | High precision and resolution;  Neutral loss;  MRM/SRM mode is more commonly used | Low collision energy;  Incomplete fragmentation;  Low full scan sensitivity | 1.Metabolic transformation of trans-emodin dianthrones a toxic substance in Polygonum multiflorum Thunb., in rat plasma, urine and feces(YANG et al., 2021; SONG et al., 2022).  2.Quantitative Analysis of Chemical Constituents in Qishiwei Zhenzhu pills. (LIANG et al., 2023).  3.Quantitative analysis of brucine and strychnine in plasma(GUO et al., 2022). |
| Q-TOF | Identification of unknown compounds;  Non-target analysis of active ingredients of TCM | High precision;  Fast acquisition rate;  Good precursor ion selectivity;  ESI/MALDI | High vacuum;  Expensive;  Significant matrix effect | 1.Identification of Shuang Huang Lian components(XU et al., 2022).  2.Active ingredients of Si-Miao-Yong-An decoction in the treatment of heart failure(LIAO et al., 2022).  3.Qualitative analysis and identification of chemical constituents of compound Fufang Xiling Jiedu capsule(CAO et al., 2021).  4.Analysis of active components in Antrodia cinnamomea(ZHANG et al., 2022). |
| IT-TOF | Non-target analysis of active ingredient;  Qualitative analysis of target compound | Simple structure;  Small size;  High ion selectivity;  Multi-stage mass spectrometry | Narrowing linear range does not allow quantification | 1.Characterize Longxue Tongluo Capsules metabolites and reveal biotransformation pathways (SUN et al., 2021).  2. Qualitative and quantitative analysis of Notoginsenoside R1 in theliver-brain-gut axis of rats(HU et al., 2022).  3. Metabolite and metabolic pathway analysis of active components in Paeoniae Radix Rubra (ZHANG et al., 2022). |
| Orbitrap | Non-target analysis and target analysis of active ingredients of TCM | High precision;  Good resolution;  Multiple scanning modes;  Good product ion selectivity | Cannot do cascading alone;  Expensive | 1.Bidens bipinnata L. active ingredient non-target analysis(WANG et al., 2022).  2. Non-targeted analysis of *Arnebiae Radix* chemical composition(ZHU et al., 2022).  3. Isolation and identification of specific binding components of Huanglian Jiedu decoction to human umbilical vein endothelial cells(LIAO et al., 2023). |
| FT-ICR | Rapid identification and qualitative analysis of active ingredients in TCM | Highest precision;  Good product ion resolution;  Suitable for multi-stage mass spectrometry | Large volume;  Expensive;  slow | 1. Metabolomics analysis of Gegenqinlian decoction on endogenous metabolites in febrile rats(LIU et al., 2020).  2. Rapid analysis of the active components of Gansuibanxia decoction(CUI et al., 2020).  3.Screening and identification of chemical constituents in Rhodiola crenulata extract that have therapeutic effects on Alzheimer disease(ZHANG et al., 2019). |
| IM | Structural identification of active ingredients、analogues、isomers of TCM | No vacuum system required;  Extremely sensitive;  Separable background noise;  Separation of structural analogues or isomers | A good ion reaction model cannot be established for a long time | 1. A pair of isoforms of plantagoside from *Plantago asiatica* L.detection and identification(GAO et al., 2022).  2. *Arnebiae Radix* unique three monosaccharides containing olefin functional groups, esters, and amino groups(SHAO et al., 2022).  3. Distinguishing nine ginsenosides involved in white ginseng and red ginseng(ZHANG et al., 2019). |
